# The Concept of Health, From Definition to Measurement and the Implications of the Reserves‐Based Model for Nursing in France

**DOI:** 10.1111/jan.16555

**Published:** 2024-10-18

**Authors:** Camille Joannès, Fanny Crozes, Michelle Kelly‐Irving, Cyrille Delpierre, Sébastien Couarraze

**Affiliations:** ^1^ EQUITY Research Team, Center for Epidemiology & Research in POPulation Health (CERPOP), UMR 1295 University Toulouse III Paul Sabatier Toulouse France; ^2^ Institute of Nursing Training Toulouse University Hospital Toulouse France; ^3^ Department of Medicine, Maieutics and Paramedicine, Faculty of Health, AGING Research Team, Center for Epidemiology & Research in POPulation Health (CERPOP), UMR 1295 University Toulouse III Paul Sabatier Toulouse France

**Keywords:** health, nursing theory, population health, research methods

## Abstract

**Aim:**

To discuss a reserves‐based model of Health, recently developed in the literature, defining Health and moving from conceptual considerations to methods of measuring Health, applicable to nursing practice and research.

**Design:**

Discursive paper.

**Methods:**

A discursive paper critically synthesising a reserves‐based model of Health for conceptualising and operationalising Health, with reference to key Health theories from nursing science and social epidemiology.

**Results:**

In the reserves‐based model of Health, Health was defined as the ability to maintain/restore physical, socioemotional, cognitive and physiological health reserves in order to adapt and self‐manage to life's challenges. Health was measured by the sum of indicators of deterioration of health reserves.

**Conclusion:**

The reserves‐based model of Health defining Health through the prism of adaptation reinforces the holistic vision of Health, appropriate to nursing practice, based on the interconnectedness of the whole person and the whole system.

**Implications for the Profession and Patient Care:**

This reserves‐based model of Health is likely to modify nursing practices. Nursing diagnosis of patient adaptation could be implemented, in order to offer support care adapted to the patient's capacity to adapt, enabling health promotion strategies to be developed. Nursing research on health reserves is a future promising direction to act on individuals' capacity to adapt.

## 1 Introduction

France is currently experiencing a major turning point in the evolution of the nursing profession and the field of nursing science. The nursing profession has been reshaped, with the autonomy of advanced practice nursing aimed at developing high‐level competencies. These include education, prevention, screening activities, technical and clinical monitoring procedures, and the ability to make prescriptions (Colson, Galfout, and Schwingrouber 2024; Décret N° 2018–629 Du 18 Juillet 2018 Relatif à l'exercice Infirmier En Pratique Avancée 2018). Nursing science has been integrated into the university setting, which implies the recognition and development of academic careers in nursing sciences (Colson et al. 2021). A new reform, expected shortly, aims to expand nursing competencies for example by emphasising key missions in prevention. It also plans to update the training curriculum, emphasising the acquisition and dissemination of research, the production of evidence‐based data and the implementation of projects focussed on community and population health. Furthermore, the reform is designed to enhance career progression and development. The current French political circumstances have led to the postponement of certain deadlines to at least 2025. Nevertheless, the nursing community has high expectations for these reforms. Health is therefore a central focus in the redefinition of nursing education and the profession. Considering this changing context, the question of Health in relation to nursing science is addressed in this discursive paper.

Health is at the core of nursing activity and, consequently, is a fundamental concept in the field of nursing research (Fawcett 1984). However, the concept of Health does raise several issues. First, it has an ambiguous meaning. What does Health actually mean? A recent scoping review found that there is a wide variety of concepts of Health, with numerous subthemes (van Druten et al. 2022). Therefore, it would be valuable to investigate the evolution of the definition of Health across various disciplines, including nursing, to identify their strengths and limitations. Second, the operationalisation of the concept of Health in care practice and research. How to measure Health? Converting this abstract concept into a measurable observation will make it easier to transpose the concept of Health into routine care. The challenge of this specific measure would be to guide care practices in a more appropriate way to suit patients' needs. Thus, it is essential to establish an operational definition of Health to move from concept to practical measurement in nursing practice and research.

In this discursive paper, we aimed to discuss a reserves‐based model of Health recently developed in the literature, by encompassing the definition of health and transitioning from conceptual considerations to methods of measurement, applicable to nursing practice and research.

## 2 Background

Over the last few decades, the concept of Health has broadened and become more complex, while simultaneously knowledge about people being cared for has increased. Within the paradigm of categorisation, the biomedical definition of Health specifying Health as the absence of disease and disability, has been most widely used in medical care and research. In this way, the concept of Health is mainly defined in terms of the absence of illness, and Health is characterised not by its own inherent attributes where ‘Health is nothing positive, it is simply not being ill’ (Herzlich 1969). This definition of Health implies identifying, isolating and treating the root cause of illnesses, as well as tackling the lethal factors involved. The nursing practices associated with this approach involve recognising the signs and symptoms that indicate the evolution of the disease, participating in the treatment administration and monitoring of the patient's condition (Lecordier 2011). However, this definition of Health has several limitations due to the categorisation of individuals as sick or not, resulting in classification biases that have long been recognised (Amzat and Razum 2014; Boorse 1977). This threshold for classifying individuals does not include the multiple symbolic values that accompany the concept of illness, which vary according to the dominant culture, civilisation and historical period (Ereshefsky 2009; King 1954). This model therefore leads to confusion between Health and medicine, the assimilation of which is too narrow to define Health.

In 1946, The World Health Organization defined Health as ‘a state of complete physical, mental and social well‐being and not merely the absence of disease or infirmity’ (World Health Organization 1946), with the aim of guiding national and global health governance. However, the utopian and unmeasurable nature of Health related to the absolute nature of the term ‘complete’, in relation to ‘well‐being’, can lead to the mistaken assumption that almost all individuals are ill (Huber et al. 2011; Larson 1996; Leonardi 2018), particularly with the ageing process. Furthermore, this definition does not consider the living conditions and the multiple factors surrounding individuals that are components of Health.

Within the paradigm of integration, Health was then broadened by considering it holistically, recognising the complexity of the environment surrounding individuals, as represented by the Dahlgren–Whitehead model (Dahlgren and Whitehead 1991). This model considers Health associated with the individual's environment (Pépin, Ducharme, and Kérouac 2017) and consisting of health determinants distributed at the individual, meso and macro levels. These health determinants are the ‘conditions in which people are born, grow, work, live, and age, and the wider set of forces and systems shaping the conditions of daily life’ (Commission on Social Determinants of Health 2008). The nursing practices associated with this approach place greater emphasis on understanding people, their values, lifestyles, history and culture, and involve cooperation within a multidisciplinary team of carers. However, while it seems promising to define the specificity of Health in a multidimensional way (mental, social and well‐being) and in relation to the external environment surrounding individuals, these definitions of Health downplay the role of individual capacities to overcome life's challenges and live well despite chronic illness or disability.

**FIGURE 1:**
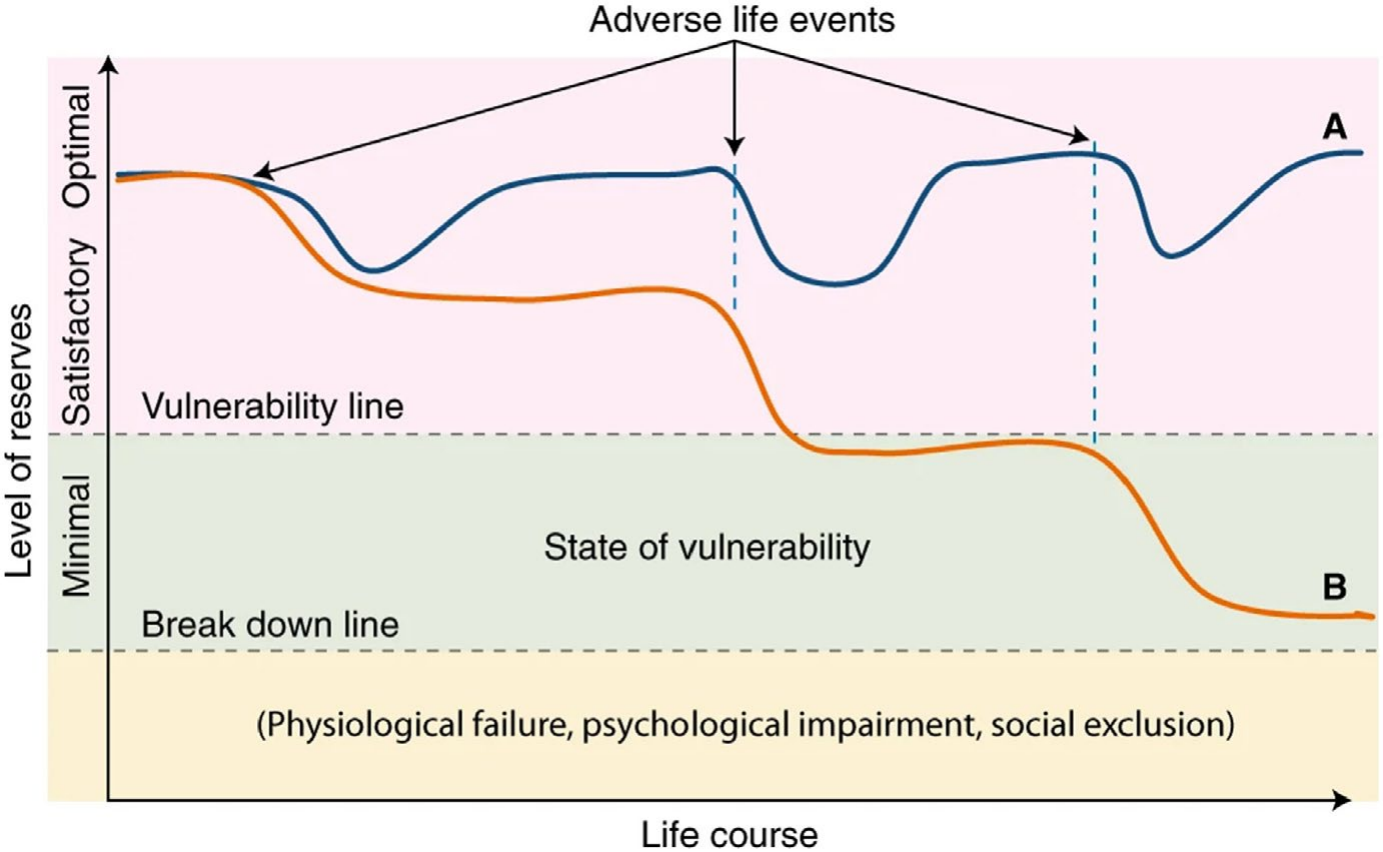
Schematic representation of the concept of reserves extracted from the study of Cullati S. and al. (Cullati, Kliegel, and Widmer 2018). The level of reserves is illustrated by the blue and orange lines. The blue line (A) represents the situation of a successful adaptation to adverse life events. The orange line (B) represents a situation of a compromised adaptation to adverse life events, leading to vulnerability.

Within the transformation paradigm with the aim of achieving harmony of body and mind, the ability of individuals to cope with their environment and to self‐manage has been recognised in the Ottawa Charter (World Health Organization 1986). In Nursing Sciences, early theories of Health and adaptation, notably Roy's adaptation model (Roy 1997), provided a robust and comprehensive theoretical framework. Roy's adaptive model posits that the individual can be conceptualised as an integrated whole, engaged in a constant interaction with the environment. This model further suggests that the individual's coping mechanisms can be enhanced through the implementation of nursing interventions (Callis 2020; Mansouri et al. 2019; Roy 2011). The nursing practices associated with this approach are moving away from the omnipotence of the carer, towards the engagement of nurses in health promotion and education, supporting individuals to solve their own health issues. In line, with Roy's adaptation model, Huber and colleagues proposed in 2011 an alternative definition of Health centred on individuals and their ability to adapt, as follows: Health is ‘the ability to adapt and self‐manage in the face of social, physical and emotional challenges’ (Huber et al. 2011). Specifically, an individual's health is understood when their psychological organisation, organism or social participation can implement a protective response to restore equilibrium in the face of social, physiological or psychological stress. This consideration of Health as the ability to cope has grown increasingly popular in recent years. However, it should be emphasised that this ability is not identical between individuals. Indeed, an individual who is born, grows up and lives in an environment with many stressors will not have the same type of adaptation during his or her life as an individual evolving in a more protective environment with more resources that can be used immediately for the needs of daily life (Cullati, Kliegel, and Widmer 2018). To address interindividual differences in adaptation, the concept of health reserves has been recently developed by Cullati and colleagues. Health reserves are latent capacities with a protective function to adapt and cope with adverse life events, and maintained by a sufficient quantity of resources (Cullati, Kliegel, and Widmer 2018). Figure 1 presents a schematic representation of health reserves as a means of adapting Health.

## 3 Overview of the Issue

This synthesis of key health theories in nursing sciences and social epidemiology to conceptualise Health has raised the interest of conceptualising it through the lens of adaptation. This perspective shifts away from solely focusing on the aetiology of diseases—examining symptoms, syndromes and systemic causes—towards a holistic conception of Health, emphasising the interrelation between individuals and their environment. The concept of health reserve has been found to be an effective way of addressing individual differences in the ‘ability to adapt and self‐manage’. The first issue is therefore to link these different conceptualisations around a single definition of Health, that is, to integrate the concept of health reserves into the definition of Health as an adaptive capacity. The second issue is to move from conceptual considerations to defining methods of measuring Health. Addressing these two issues would then provide a Health framework applicable to nursing practice and research. This discursive paper will present and discuss a reserves‐based model of Health recently developed in the literature (Joannès et al. 2023) that addressed these issues.

## 4 Results

### 4.1 Definition of Health in the Reserves‐Based Model

In the light of the conceptual evolution in the definitions of Health, the reserves‐based model of Health has defined Health through the prism of adaptation (Huber et al. 2011) and further developed it by incorporating the concepts of reserves developed by Cullati and colleagues (Cullati, Kliegel, and Widmer 2018). The definition of Health used was the individual's ability to maintain or restore health reserves in order to adapt and self‐manage to social, physical and emotional challenges (Joannès et al. 2023). Health reserves have been identified as physical, socioemotional, cognitive (Ben‐Shlomo 2013) and physiological (Lu, Pikhart, and Sacker 2019). Table 1 presents the description of each health reserve.

**TABLE 1:** Description of health reserves and example of indicators of measurement.

Health reserve		Examples of indicators of deteriorating health reserves
Physical reserve	Refers to an individual's intrinsic physical or functional capacity of body functions, accumulated over a lifetime, to carry out physical activities. It aims to maintain functional integrity and contributes to an adaptive response to environmental physical challenges (Kuh et al. 2014)	Chronic pain or Chronic Widespread Pain (Jones et al. 2007; Jones, Power, and Macfarlane 2009) Grip strength (Sayer and Kirkwood 2015) Standardised physical mobility tests (Soubra, Chkeir, and Novella 2019)
Socio‐emotional reserve	Characterises the intrinsic ability, built up throughout life and influenced by social and cultural factors, to cope with an emotional and social stressor (Ben‐Shlomo, Cooper, and Kuh 2016; Lu, Pikhart, and Sacker 2019)	Clinical Interview Schedule (CIS‐r) (Lewis et al. 1992) Malaise inventory (Scully 2014) Schedule for Clinical Assessment in Neuropsychiatry (SCAN) (Wing et al. 1990) Patient Health Questionnaire (PHQ) (Kroenke, Spitzer, and Williams 2001) Hamilton Anxiety and Depression Scale (Ham‐A and Ham‐D) (Maier et al. 1988) Other validated screening instrument assessing psychological distress
Cognitive reserve	Corresponds to the ‘level of protection against the clinical manifestations of neurological damage’, that is, the capacity accumulated throughout life to maintain and improve cognitive functioning in order to delay its decline due to ageing (Stern 2009)	The Trail Making test A and B (Ciolek and Lee 2020) Cognitive Reserve Index questionnaire (Nucci, Mapelli, and Mondini 2012) Montreal Cognitive Assessment (MoCA) test (Nasreddine et al. 2005) Other validated cognitive assessment
Physiological reserve	Related to the concept of allostasis, an active process by which the body attempts to maintain normal physiological regulation in response to stress (Sterling and Eyer 1988) and avoid physiological imbalances across systems	Allostatic load (McEwen and Stellar 1993) Ageing scores Other biological scores (Karimi et al. 2019)

### 4.2 Reserves‐Based Model Method for Measuring Health

This consideration of Health through adaptation means that if the presence of adaptation (*p*) implies optimal health (*q*), then impaired health (not *p*) necessarily implies compromised adaptation (not *q*), and vice versa. Given the complexity of measuring adaptation itself, the reserves‐based model chose to measure it through its contrapositive, that is, a compromised adaptation, also called accommodation (Frisancho 1993). It has been assumed that accommodation generates a cost on health reserves. This cost on health reserve could be observable in midlife, after the build‐up phase during the youth and before the natural decline phase of later life, as can be expected with ageing and the progressive loss of functional and cognitive abilities as illustrated by the Strachan–Sheikh Model (Blane et al. 2013; Kuh and Ben‐Shlomo 2004). The assumption is that the cost on health reserves can be then measured by indicators of health reserve deterioration. According to the definition adopted, four health reserves were retained, that is, physical, socioemotional, cognitive and physiological reserves. Indicators of deteriorating health reserves were considered to be clinical symptoms or validated measures of chronic wear and tear corresponding to health reserves. A list of nonexhaustive indicators of deteriorating health reserves that could be available and used in care practice to measure Health has been provided (Table 1).

Once a set of indicators has been selected, this reserves‐based model proposed to combine them by summing them to construct a measure of overall Health. Assuming that each indicator had the same weight as the others, each indicator was given a uniform weight. The overall Health measure can then be classified into three categories: ‘Optimal/Medium/Impaired’. The ‘Optimal’ corresponds to the absence of values taken by indicators indicating a potential health risk or wear and tear. The ‘Medium’ corresponds to the presence of a value taken by an indicator indicating a potential health risk or wear and tear. The ‘Impaired’ category corresponds to the presence of two or more values taken by indicators indicating a potential health risk or wear and tear.

### 4.3 Application of the Reserves‐Based Model Method to Measure Health Within a Cohort

An application of the reserves‐based model was performed using data of 9377 subjects from the UK birth cohort *National Child Development Study* (NCDS 58). The data used were those collected when the individuals were aged between 44 and 45, through a biomedical survey (blood and saliva samples and anthropometric measurements) and a home clinical assessment. A measure of overall Health in middle adulthood was obtained with the identification of indicators of deterioration in health reserves. The indicators selected were *allostatic load* (AL) for physiological reserve, *chronic widespread pain* (CWP) for physical reserve and the *clinical interview schedule‐revised* (CIS‐r) for socioemotional reserve. No indicator of cognitive reserve was available in the data set. Each indicator was reformatted so that their categories varied from 0 to 1. The overall measure of Health was constructed by summing the previously selected indicators. The overall health measure was then categorised into three groups: ‘Optimal/Medium/Impaired’ based on the hypothetico‐deductive methodology developed previously: the ‘Optimal’ category corresponded to the absence of health risk value taken by AL, CWP and CIS‐r; the ‘Medium’ category corresponded to the presence of one health risk value taken by AL, CWP and CIS‐r; the ‘Impaired’ category corresponded to the presence of two or more values taken by AL, CWP and CIS‐R. Table 2 illustrates the construction of the overall health measure according to the categories of each selected indicator.

**TABLE 2:** Construction of the overall health measure according to selected indicators.

Selected indicators				Overall health
Allostatic load	Clinical Interview Schedule revised	Chronic Widespread Pain		
Low	No mental disorder	No Chronic Widespread Pain	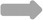	**Optimal**
Medium	No mental disorder	No Chronic Widespread Pain	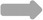	**Medium**
High	No mental disorder	No Chronic Widespread Pain		
Low	Common mental disorders	No Chronic Widespread Pain		
Low	No mental disorder	Chronic Widespread Pain		
Medium	Common mental disorders	No Chronic Widespread Pain	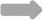	**Impaired**
Medium	No mental disorder	Chronic Widespread Pain		
High	Common mental disorders	No Chronic Widespread Pain		
Low	Common mental disorders	Chronic Widespread Pain		
High	No mental disorder	Chronic Widespread Pain		
Medium	Common mental disorders	Chronic Widespread Pain		
High	Common mental disorders	Chronic Widespread Pain		

*Note*: The grey colour of the initial two rows serves to differentiate the column headings and sub‐headings from the subsequent results. The grey arrows indicate the category to which each row of results in the right‐hand column belongs.

The criterion validity of the overall Health measure was examined by modelling the relationship between the overall Health measure and subsequent mortality as well as later self‐rated health, first using logrank tests or Chi square, then logistic regressions or Cox proportional regression, as appropriate. Statistical modelling found that individuals with an impaired overall Health were at greater risk of dying or reporting poor perceived health over a 14‐year period following the initial overall Health measure.

The robustness of the method was assessed using comparative analyses. A further grouping of the general health measure indicated that the observed results did not depend on the categorisation of the general health measure. Another choice of health reserve indicator reported that the observed results were not biased by the variables constituting the overall Health measure. Random subsamples of the data using a negative control variable were set up and revealed the results observed were not biased by the data of the initial sample.

## 5 Discussion

### 5.1 Summary of the Findings and Comparison With Other Health Models

The reserves‐based model has defined Health as the individual's ability to maintain or restore health reserves in order to adapt, and the measurement of Health was based on the identification and summation of indicators of deterioration in health reserves. The measure created from this model based on reserves was found to be a good predictor of subsequent poor perceived health and mortality.

The reserves‐based model is in line with holism in nursing sciences (Dossey et al. 2015). Indeed, the reserves‐based model considers not only the physical aspects of health but also the emotional, mental and social dimensions of the individual and recognises that these they are interconnected and that imbalances in one dimension can affect the others, which has an impact on the overall measure of health of the reserves‐based model. However, the reserves‐based model involves dimensionality with the consideration of the socioemotional, physiological, physical, cognitive reserves, which may diverge from holism that considers health from a global perspective. This fragmentation of Health arises from the objective of operationalising the concept of Health for quantitative measurement. Despite this initial fragmentation, the reserves‐based model ultimately achieves reunification as the dimensions are reconsidered collectively through the aggregation of each reserve's indicators, leading to a comprehensive measurement of health in its holistic sense.

The reserves‐based model is also in line with the adaptation model proposed by Roy, which postulates that individuals adapt to environmental influences, or stimuli, using innate or acquired adaptation mechanisms, which are biological, psychological and social (Roy 1997). Roy's Adaptation Model implies that the individual adapts to changes in the environment or responds to stimuli using innate or acquired adaptation mechanisms, which are biological, psychological and social. This model is applied in nursing practice whenever a patient suffers from an illness for the first time or experiences a difficulty in their state of health, or faces the end of their life, to give them the means to act and achieve positive results. However, while the reserves‐based model studied physical, psychosocial, cognitive and physiological reserves, Roy's model considers modes of adaptation to be ‘physiological’, ‘self‐concept’, ‘role function’ and ‘interdependence’. These similarities and differences with the model proposed by Roy, on the one hand, reinforce the approach of the reserves‐based model in the field of nursing and, on the other hand, open up discussions on its application in the nursing process, particularly when assessing patient's behaviour and reaction to stimuli, and the nursing response in return.

### 5.2 Limitations

The reserves‐based model of Health has its limitations, notably related with the measurement of Health itself.

A first limitation is related to the thresholds chosen. In the current application, the allostatic load indicator used in the construction of the measurement of Health is categorised as low/medium/high based on its statistic distribution within the UK study cohort. The question then arises as to its use in nursing practice if there is no validated ‘clinical’ threshold for the indicators chosen. Furthermore, the thresholds for defining ‘optimal’, ‘medium’ and ‘impaired’ Health are of major consequences for nursing practice: a change in an individual's health status from ‘optimal’ to ‘medium’ Health is of less concern from a change in health status from ‘medium’ to ‘impaired’ Health. These issues deserve to be further investigated.

A second limitation is related to the nonexhaustive, and therefore modifiable, list of indicators of health reserve deterioration. This list must be improved, particularly for the use of this concept in nursing practice. It would be of interest to compare the indicators used in the reserves‐based model with those used in population studies to measure human Health (OECD Publishing 2023) or those related to quality of life, such as the SF‐36 (Ware 2000), or frailty, which are used in research on healthy and successful ageing (Fuellen et al. 2019; Lara et al. 2013; Lu, Pikhart, and Sacker 2019). This list must also be developed when an indicator cannot be measured, as in the present application where the cognitive reserve was not measured as no corresponding indicator was identified. This implied a lack of precision in the validation of the measurement of overall Health with a possible underestimation of the results observed with mortality and perceived health.

A third limitation is related to the absence of differentiated weighting of indicators in the measurement of overall Health. This assumes that each indicator corresponds to a health reserve and that these reserves theoretically play an equivalent role in Health. Defining the precise role that each reserve could have on the others and on Health is a complex issue when choosing the weighting to be assigned. This reserves‐based model, which is relatively simple via the sum of indicators, produces a synthetic measure that has been shown to capture a common variance between its various components.

A last limitation relates to the transferability of the measure of Health. The measure of Health studied was a cross‐sectional measure in midadulthood, supposed to reflect the overall Health of the individual. It first raises the question of how this non‐prospective measure of Health can be inferred from Health at other ages. The choice of this cross‐sectional measure is based both on Krieger's ecosocial theory (Krieger 2001), which postulates that Health at a given age is the consequence of a life‐course process affected by all the past experiences, and also on the Strachan–Sheikh Model (Kuh and Ben‐Shlomo 2004), which postulates that health follows a trajectory resulting from a build‐up followed by a decline phase related to advanced age. However, the measurement of Health in midadulthood between these two phases is only valid for cognitive and motor functions, which follow these trajectories, but not for well‐being, which has a U‐shaped trajectory over the life course (Blanchflower 2021). This raises the question of the appropriate period of life for measuring socioemotional reserve. Further research is required in nursing science to analyse whether repeated Health measurements provide better information about an individual's holistic Health.

The United Kingdom is home to a distinctive series of birth cohort studies, which provide a unique opportunity to examine health status across the life course and to compare health outcomes with those of non‐French‐speaking Western health systems. France has a number of databases, including EPP, Gazel, E3N, Paquid, EVA and SNDS. However, it is unclear whether these databases contain sufficiently detailed information to measure health using the reserves‐based model. Furthermore, it is uncertain whether nurses can access or collect this information on a routine basis. Discussion and evolution are expected to prioritise the collection of and access to this data.

### 5.3 Perspectives

This set of limitations opens avenues for discussion between diverse professionals from nursing sciences, medicine, public health, social epidemiology, psychology and philosophy of sciences about the different health models and suggests a more in‐depth conceptual analysis to improve the reserves‐based model in relation to the theory of adaptation in nursing care. From a nursing science and practice perspective, consideration should be given to translating the reserves‐based model at individual level rather than population level. Indeed, the global health measure is currently validated with probability analyses comparing population groups with others, producing averaged results of individual data. The current composite measure of Health, itself based on composite measures such as allostatic load composed of a sum of biomarkers, and the thresholds for these measures initially defined/validated on populations with their own characteristics, requires applied research to help identify and implement pragmatic ways of operationalising this concept in nursing care.

Furthermore, nursing research on the various dimensions that make up these reserves and to study health reserves throughout life is a future promising direction. This could enable to examine the way in which health reserves are built up, maintained and activated over the life course. This could lead to identify factors likely to affect ‘the ability to adapt and self‐manage’, and specifically those are likely to ‘restore’ health reserves, thereby contributing to improve health. The identification of the determinants of positive health would make it possible to develop interventions, involving nurses, to enhance individuals' ability to adapt, that is, to act on resilience in health.

Given that health promotion is one of the nursing profession's core missions, the reserves‐based model would enable a redefinition of the contours of these missions to carry out potentially more relevant actions according to the forthcoming new French nursing decree. Healthcare practices aligned with the reserves‐based model may encompass nursing assessments and diagnosis of a patient's health adaptation, considering their age, sex and levels of health reserve, using indicators of their deterioration through health wear and tear. Through the prism of wear and tear, this approach will make it possible to measure the patient's adaptation to the health consequences of their life course, enabling tailored supportive care based on their specific circumstances. Healthcare professionals' and patients' understanding of the meaning of health reserves will encourage patients' active participation and empowerment, and help avoid misunderstandings in practice (Song and Kong 2015). In addition, the importance of accounting for inequalities in adaptation between individuals enables an equitable approach of care that can addresses social health inequalities.

This approach based on the reserves model can lead to an evolution in practices public health for nursing in France. An example illustrating this evolution is the HIV screening (Leblanc et al. 2018). Prior to 2012, HIV screening in France was nontargeted and performed by doctors in emergency departments for patients with symptoms related to HIV, following a ‘population approach’. However, this method was insufficient for early infection detection. A few years later, screening practices evolved into a ‘high‐risk strategy’, with nurses conducting targeted HIV screening based on individual patient characteristics. This approach could further develop into a ‘vulnerable population approach’, considering individuals' health reserves and implementing targeted actions based on their health adaptation with HIV diagnosis. The paradigmatic evolution of health through adaptation calls for an evolution of nursing practices, which could be taken up by advanced practice nurses with support from the current healthcare system in France in prevention and health promotion, although their political agenda prioritisation is not emphasised. Furthermore, the measures do not exhaust the possibilities for enabling France to benchmark health care outcomes with those of non‐Francophone Western healthcare systems. At a time when public policies are increasingly geared towards preventive measures, and when professions are evolving, the way to (re)think about Health is now crucial.

## 6 Conclusion

The conception of Health through the prism of a coping mechanism reverses the traditional negative way of considering Health as the presence of disease. This new positive and community‐based approach, integrating a holistic dimension of Health, has a strong message based on the identification of factors favouring self‐fulfilment (Fowokan et al. 2023). This paradigmatic evolution is not just a conceptual change, it is also likely to profoundly modify the healthcare system through its innovative actions. The role of the various healthcare players, including nurses, can be significantly modified and strengthened by the missions entrusted to them. This broader, and therefore less medical, definition of health strengthens the role of other professions, first and foremost nurses, whose mission is to take into account the patient as a whole, and also to promote health, from the patient's point of view to the person in their environment.

## Conflicts of Interest

The authors declare no conflicts of interest.

### Peer Review

The peer review history for this article is available at https://www.webofscience.com/api/gateway/wos/peer‐review/10.1111/jan.16555.

## Data Availability

The authors have nothing to report.
